# MicroRNA-1270 Inhibits Cell Proliferation, Migration, and Invasion via Targeting IRF8 in Osteoblast-like Cell Lines

**DOI:** 10.3390/cimb44030077

**Published:** 2022-03-01

**Authors:** Eric Gustavo Ramírez-Salazar, Erika Victoria Almeraya, Tania Valentina López-Perez, Zacarías Jiménez-Salas, Nelly Patiño, Rafael Velázquez-Cruz

**Affiliations:** 1 Consejo Nacional de Ciencia y Tecnología (CONACYT)-Instituto Nacional de Medicina Genómica (INMEGEN), Mexico City 14610, Mexico; eramirez@inmegen.gob.mx; 2 Laboratorio de Genómica del Metabolismo Óseo, Instituto Nacional de Medicina Genómica (INMEGEN), Mexico City 14610, Mexico; erikaalmeraya@yahoo.com.mx (E.V.A.); valecita_vale@hotmail.com (T.V.L.-P.); 3 Centro de Investigación en Nutrición y Salud Pública, Universidad Autónoma de Nuevo León, Monterrey 64460, Mexico; zacarias.jimenezs@uanl.mx; 4 Unidad de Citometría de Flujo (UCiF), Instituto Nacional de Medicina Genómica (INMEGEN), Mexico City 14610, Mexico; lnpatino@inmegen.gob.mx

**Keywords:** bone metabolism, gene regulation, microRNA, osteoblast, osteoclast, osteoporosis

## Abstract

Osteoporosis (OP) is the most common bone disease affecting elderly individuals. The diagnosis of this pathology is most commonly made on the basis of bone fractures. Several microRNAs (miRNAs/miRs) have been identified as possible biomarkers for the diagnosis and treatment of OP. miRNAs can regulate gene expression, and determining their functions can provide potential pharmacological targets for treating OP. A previous study showed that miR-1270 was upregulated in monocytes derived from postmenopausal women with OP. Therefore, the present study aimed to uncover the role of miR-1270 in regulating bone metabolism. To reveal the mechanism underlying the regulatory effect of miR-1270 on interferon regulatory factor 8 (IRF8) expression, luciferase assay, reverse transcription-quantitative PCR, and Western blot analysis were performed. The results suggest that miR-1270 could regulate the mRNA and protein expression levels of IRF8 by directly binding to its 3′-untranslated region. The effects of miR-1270 overexpression and IRF8 silencing on cell proliferation, migration, and invasion were also evaluated. To the best of our knowledge, the current study was the first to support the crucial role of miR-1270 in bone metabolism via modulation of IRF8 expression. In addition, miR-1270 overexpression could attenuate human osteoblast-like cells’ proliferation and migration ability.

## 1. Introduction

Osteoporosis (OP) is the most common bone disease, recognized as a major public health issue due to increased life expectancy [1,2,3]. The development of OP is closely associated with genetic and environmental factors [2,4,5]. The prevalence of OP is increased in elderly and postmenopausal women, who suffer from fractures that diminish their quality of life [6,7,8]. Moreover, OP frequently goes undiagnosed until a fracture occurs [1,3]. Bone tissue is very dynamic and complex, and its metabolism is modulated via a life-long process of demodulation regulated by osteoblasts and osteoclasts [9,10]. Therefore, studying these well-differentiated cell types is a critical aspect of understanding the remodeling process, and has been accomplished using primary cultures and established cell lines [11,12,13]. In tight coordination, osteoblasts and osteoclasts regulate bone resorption and the cyclic process of bone formation [12,14,15]. Disruption of this equilibrium may lead to several pathological conditions such as osteopenia and OP, varying degrees of the same disease caused by untargeted and excessive bone resorption, and reduced bone formation [4,6,16]. Since osteoclasts and osteoblasts regulate bone remodeling, the crosstalk between those two cell types is considered crucial [13,17,18]. Emerging evidence has suggested that mechanisms to restore the balance of bone formation and resorption are considered a practical therapeutic approach for treating women with postmenopausal OP [5,19,20]. However, the currently available treatment protocols mainly inhibit osteoclast activity (resorption) [21,22]. It is well known that osteoclasts can regulate osteoblast activity either by direct interaction or indirectly by the secretion of cytokines [23]. Other studies have demonstrated the presence of miRNAs in body fluid, such as serum, and transported in extracellular vesicles or exosomes, unveiling their key function as extracellular signals between cells. However, it is unclear whether the osteoclast–osteoblast communication can be accomplished via other efficient ‘paracrine’ ways [24,25].

Therefore, uncovering the molecular mechanisms underlying osteogenic bone formation is of great importance for identifying novel osteogenic drug targets. Several studies during the last two decades have identified microRNAs (miRNAs/miRs) associated with osteoclast differentiation, such as miR-548x-3p, miR-708-5p, miR-133a, miR-422a, and miR-148a [26,27,28]. A previous study investigating the miRNA profile of postmenopausal women with OP showed that miR-1270 was upregulated, and IRF8 (a key regulator of bone metabolism) was downregulated in circulating monocytes [29,30]. Bioinformatics analysis and literature screening also supported that IRF8 was the most promising target gene of miR-1270 [29]. It has also been reported that miR-1270 is associated with several types of cancer [12,31,32] and plays a vital role in the innate immune system by maintaining the physiological type I IFN [30]. However, the effect of miR-1270 on bone metabolism has not been previously investigated. Therefore, the present study aimed to evaluate the regulatory effect of miR-1270 on the expression of IRF8 in human osteoblast-like cell lines by performing luciferase activity, proliferation, migration and invasion assays, and target gene expression analysis.

## 2. Materials and Methods

*Cell lines and stable transfection*. The human osteosarcoma-derived cell lines U2OS and SaOS-2 were obtained from the American Type Culture Collection and cultured in McCoy’s 5A medium (cat. no. M4892; Sigma-Aldrich; Merck KGaA, Darmstadt, Germany) supplemented with 10 and 15% heat-inactivated FBS (cat. no. S165H; Biowest, Riverside MO, USA), respectively. All cell cultures were maintained at 37 °C in a humidified atmosphere of 5% CO_2_. The sequence of pre-miR1270 was sub-cloned into the pcDNA6.2 plasmid using the BLOCK-iT Pol II miR RNAi Expression Vector kit with EmGFP (cat. no. K493600; Invitrogen; Thermo Fisher Scientific, Inc., Carlsbad, CA, USA) according to the manufacturer’s instructions. The cell lines were transfected with 10 μg pcDNA6.2-miR-1270 plasmid using Lipofectamine^®^ 2000 transfection reagent (cat. no. 11668019, Invitrogen; Thermo Fisher Scientific, Inc., Carlsbad, CA, USA). The green fluorescent protein (GFP) reporter expression was analyzed 48 h after transfection under a fluorescence microscope (EVOS FL; Thermo Fisher Scientific, Inc.). Antibiotic selection established stably transfected cell lines following cell treatment with 10 ng/μL blasticidin (cat. no. 15205; Sigma-Aldrich; Merck KGaA, Darmstadt, Germany) for two weeks before using them for further analysis. 

*Luciferase activity assays*. The pMir-Target plasmid encompassing the human wild-type (wt) IRF8 3′-untranslated region (pMir-Target-IRF8_3′-UTR wt) was purchased from OriGene Technologies, Inc., Rockville, MD, USA (cat. no. SC214035). The mutated version of the binding sequence of miR-1270 on IRF8 3′-UTR (pMir-Target-IRF8_3′-UTR mut) was generated using the QuickChange II site-directed mutagenesis system (cat. no. 200524; Stratagene; Agilent Technologies, Inc., Santa Clara, CA, USA) following the manufacturer’s instructions. Cells were seeded in a 12-well plate at a density of 3 × 10^5^ cells/well. The next day, cells were transfected for 6 h with 600 ng pcDNA6.2-miR-1270 and pMirTarget-IRF8-wt or pMir-Target-IRF8-out using Lipofectamine^®^ 2000 transfection reagent (cat. no. 11668019, Invitrogen; Thermo Fisher Scientific, Inc., Carlsbad, CA, USA) in serum-free Opti-MEM I Reduced-Serum Medium (cat. no. 31985062; Gibco; Thermo Fisher Scientific, Inc., Grand Island, NY, USA) according to the manufacturer’s instructions. A plasmid with an inhibitory sequence for miR-1270 (pcDNA6.2-Inh-miR-1270) was used to demonstrate binding specificity. The pmiR-reporter-β-galactosidase plasmid was used as an internal control. Luciferase activity was measured using the Dual-Light Luciferase & β-Galactosidase Reporter Gene Assay System (cat. no. T1003; Invitrogen; Thermo Fisher Scientific, Inc., Carlsbad, CA, USA) at 48 h following cell transfection, according to the manufacturer’s recommendations. The assays were performed in triplicate in three independent experiments.

*Reverse transcription-quantitative PCR (RT-qPCR)*. According to the manufacturer’s instructions, total RNA was isolated from 6–8 × 10^6^ stably transfected and non-transfected cells using TRIzol^®^ reagent (Invitrogen; Thermo Fisher Scientific, Inc., Carlsbad, CA, USA). Briefly, cells were washed twice with 1X PBS, and then 600 μL TRizol^®^ reagent was added to the cell monolayer. Subsequently, cells were collected with a cell scraper, transferred into a 1.6 mL tube, supplemented with 200 μL chloroform, and incubated for 5 min at room temperature, followed by centrifugation at 12,000× *g* for 20 min at 4 °C. The aqueous phase was then collected in a new tube, and 1.5 volumes of absolute ethanol were added into the tube, followed by incubation at −20 °C for 20 min. Following centrifugation at 12,000× *g* for 20 min at 4 °C, the RNA pellet was washed with 750 μL 70% ethanol and then resuspended in 50 μL molecular-biology-grade water. Subsequently, total RNA (1 μg) was reverse transcribed into cDNA using the MicroRNA Reverse Transcription kit (cat. no. 4366596; Applied Biosystems; Thermo Fisher Scientific, Inc., Foster City, CA, USA). qPCR was performed using TaqMan^®^ assay (cat. no. 002807; Applied Biosystems; Thermo Fisher Scientific, Inc., Foster City, CA, USA). For IRF8 expression analysis, cDNA was synthesized using 1 μg total RNA as a template, and dT oligos (cat. no. SO131, Thermo Fisher Scientific, Inc., Carlsbad, CA, USA), using the High-Capacity cDNA Reverse Transcription kit (cat. no. 4368814; both from Applied Biosystems; Thermo Fisher Scientific, Inc., Foster City, CA, USA). qPCR was performed using specific primers for IRF8 and the SYBR Select Master Mix kit (cat. no. 4472897; Applied Biosystems; Thermo Fisher Scientific, Inc., Foster City, CA, USA). The miR-1270 expression levels were normalized to U6 snRNA (cat. no. 001973; Applied Biosystems; Thermo Fisher Scientific, Inc., Foster City, CA, USA), and IRF8 to GAPDH expression. All PCR reactions were performed on the QuantStudio 7 Flex system (Applied Biosystems; Thermo Fisher Scientific, Inc., Foster City, CA, USA) using standard thermocycling conditions of 50 °C for 2 min, 95 °C for 10 min, followed by 50 cycles at 95 °C for 15 s and 60 °C for 1 min. The expression levels were determined using the 2^−ΔΔCq^ method. The RT-qPCR assays were performed in triplicate in three independent reactions.

*Western blot analysis*. Protein lysates were extracted from 6–8 × 10^6^ cells using RIPA buffer (cat. no. 20–188; MilliporeSigma, Burlington, MA, USA) supplemented with protease inhibitor cocktail (cat. no. P8340; MilliporeSigma, Burlington, MA, USA). Briefly, cells were washed twice with 1× PBS, and 500 μL RIPA buffer with protease inhibitors was added onto the cell monolayer. Subsequently, cells were collected with a cell scraper and transferred into a 1.6 mL tube followed by centrifugation at 8000× *g* for 10 min at 4 °C. The supernatant was divided into 100 μL aliquots and stored at −80 °C until use. For protein analysis, three independent cultures were prepared. Proteins were separated by 10% SDS-PAGE and transferred onto PVDF membranes. Following blocking, the membranes were incubated with primary antibodies against IRF8 (1:1000; cat. no. ab28696) and GAPDH (1:5000; cat. no. ab8245), both from Abcam. Subsequently, the membranes were incubated with anti-rabbit (cat. no. ab205718) and anti-mouse (cat. no. ab6728; both from Abcam, Cambridge, UK) HRP-conjugated secondary antibodies, respectively. Chemiluminescence signal was developed using the Amersham ECL Prime Western Blotting System (cat. no. RPN2232; Amersham; Cytiva) and detected using the GelDoc™XR Plus system (Bio-Rad Laboratories, Inc., Hercules, CA, USA). Each assay was performed in triplicate in three independent experiments. 

*Proliferation assay*. Cell proliferation was assessed using an MTS assay (Promega Corporation). Briefly, 3 × 10^4^ stably transfected and non-transfected cells were seeded into a 24-well plate and incubated for 0, 24, 48, 72, and 96 h. Finally, the absorbance at a wavelength of 490 nm was measured in each well. Each assay was performed in triplicate in three independent experiments.

*Migration and invasion assays*. Cell invasion and migration abilities were evaluated using Transwell chambers (pore size, 8 μm) coated or not with Matrigel (Corning, Inc., Corning, NY, USA), respectively. Briefly, 5 × 10^4^ cells in 100 μL FBS-free medium were seeded into the top compartment of the chamber, while the bottom one was supplemented with 1 mL standard medium. Following incubation at 37 °C overnight, migrated or invasive cells were fixed, stained with 0.2% crystal violet solution, and counted in six randomly selected membrane areas under a light microscope. Each Transwell assay was carried out in triplicate in three independent experiments.

*Statistical analysis*. All statistical analyses were performed with a Mann–Whitney test using GraphPad Prism version 6.0 software (GraphPad Software, Inc., San Diego, CA, USA; www.graphpad.com (accessed on: 21 July 2021)). Data are expressed as the mean ± SD as indicated. *p* < 0.05 was considered to show a statistically significant difference.

## 3. Results

**Figure 1:**
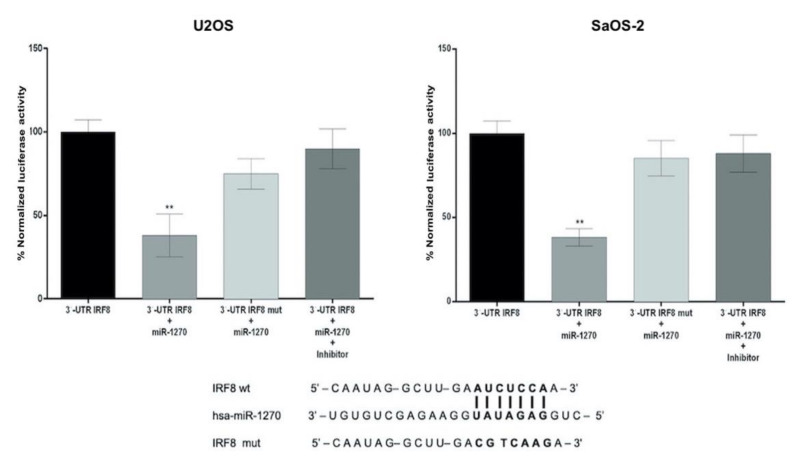
miR-1270 directly targets IRF8 3′-UTR. The binding sequence of miR-1270 on IRF8 3′-UTR is shown. The luciferase activity was measured in cells co-transfected with wild-type or mutant pmiR-Target-IRF8_3′-UTR and miR-1270 mimics/inhibitor. Data are expressed as the mean ± SD from three independent experiments. Non-parametric Mann–Whitney U test was performed. ** *p* < 0.01. miR-1270, microRNA-1270; IRF8, interferon regulatory factor 8; 3′-UTR, 3′-untranslated region.

*miR-1270 binds to IRF8 3′-UTR*. Firstly, the current study aimed to investigate the binding capacity of miR-1270 on IRF8 3′-UTR. Therefore, SaOS-2 and U2OS cells were stably co-transfected with miR-1270 and wild type (wt) or mutant (mut) IRF8 3′-UTR. Luciferase activity assay results showed that the luciferase activity was decreased in cells co-transfected with 3′-UTR-wt and miR-1270 mimics, but not in those transfected with 3′-UTR-mut. Additionally, the luciferase activity was rescued in cells transfected with miR-1270 inhibitor (Figure 1).

**Figure 2:**
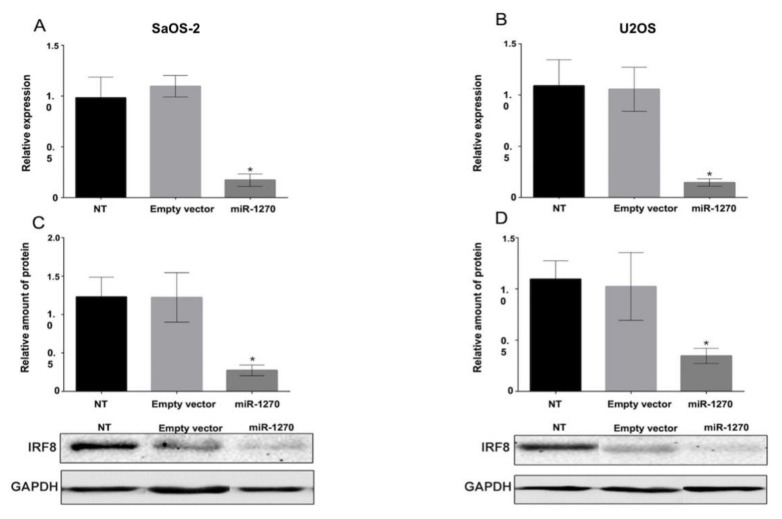
MicroRNA-1270 overexpression reduces the mRNA and protein expression levels of IRF8. Reverse transcription-quantitative PCR analysis was carried out to determine the mRNA expression levels of IRF8 in (**A**) SaOS-2 and (**B**) U2OS cells using SYBR Green technology. GAPDH was used as a reference gene. Densitometric analysis of IRF8 protein expression levels in transfected and non-transfected (**C**) SaOS-2 and (**D**) U2OS cells is shown. Data are expressed as the mean ± SD from three independent experiments. Non-parametric Mann–Whitney U test was performed. * *p* < 0.05. IRF8, interferon regulatory factor 8.

*miR-1270 promotes IRF8 downregulation*. It has been reported that osteoclasts interact with osteoblasts [17,18,25]. Therefore, herein, miR-1270 was overexpressed in osteoblast-like cells. Since miR-1270 was overexpressed in circulating monocytes (osteoclast precursors), which in turn could interact with osteoblasts, the current study hypothesized that miR-1270 could act as a gene regulator in osteoblasts. To address this, the regulatory effect of miR-1270 on IRF8 expression was investigated. The expression levels of IRF8 were quantified in cells stably transfected with miR-1270 mimics. qPCR analysis revealed that miR-1270 overexpression could reduce the mRNA expression levels of IRF8 in the osteoblastic cell lines SaOS-2 and U2OS (Figure 2A,B). Furthermore, miR-1270 overexpression decreased the protein levels of IRF8 in both osteoblastic cell lines (Figure 2C,D). The above findings indicated that miR-1270 could modulate the transcriptional and translational expression of IRF8.

**Figure 3:**
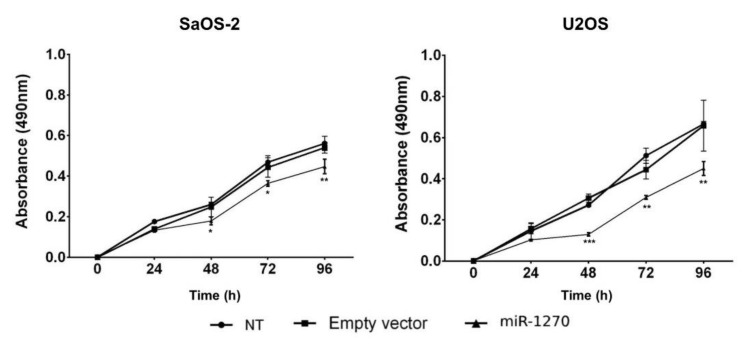
miR-1270 overexpression attenuates cell proliferation. The cell proliferation ability was assessed in non-transfected or transfected SaOS-2 and U2OS cells with miR-1270 mimics or empty vectors using MTT assay. Data are expressed as the mean ± SD from three independent experiments. Non-parametric Mann–Whitney U test was performed. * *p* < 0.05, ** *p* < 0.01, and *** *p* < 0.001. miR-1270, microRNA-1270.

*miR-1270 attenuates osteoblast proliferation, migration, and invasion*. Stable transfection of the osteoblast-like cell lines SaOS-2 and U2OS with miR-1270 mimics allowed the investigation of the effects of miR-1270 on the proliferation, migration, and invasion abilities of bone-derived cells. The results demonstrated that miR-1270 significantly decreased the proliferation rate in both cell lines (Figure 3). 

**Figure 4:**
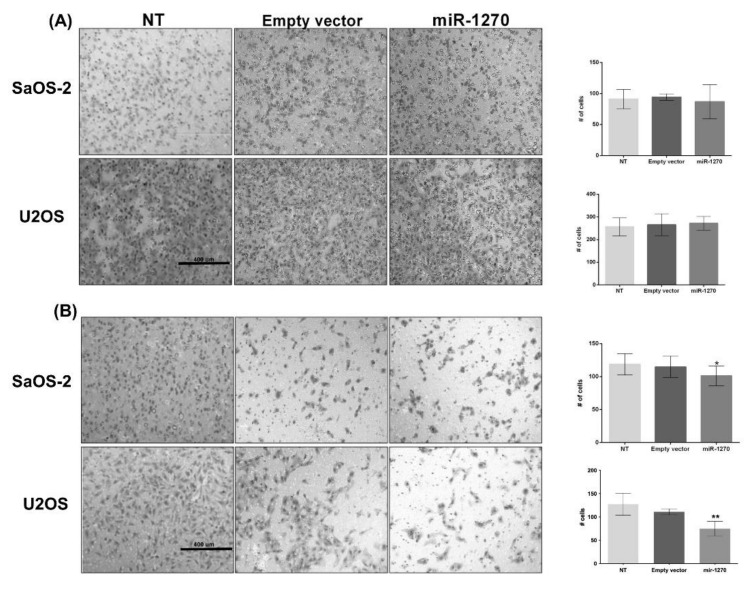
miR-1270 overexpression attenuates the migration and invasion of human osteosarcoma cell lines. The migration and invasion abilities of non-transfected or transfected cells with miR-1270 mimics or empty vectors are shown. (**A**) Representative images of transfected and non-transfected SaOS-2 and U2OS cell migration are shown. Following cell migration assay and cell staining with crystal violet, images of the migrated cells were captured under a microscope (magnification, 10×). (**B**) Representative images of transfected and non-transfected SaOS-2 and U2OS cell invasion are shown. Following cell invasion assay and cell staining with crystal violet, images of the invaded cells were captured under a microscope (magnification, 10×). Data are expressed as the mean ± SD from three independent experiments. Non-parametric Mann–Whitney U test was performed. * *p* < 0.05 and ** *p* < 0.01. miR-1270, microRNA-1270.

Furthermore, the effects of miR-1270 on the migration and invasion capabilities of the osteoblastic cell lines were evaluated. As shown in Figure 4A, there was no statistically significant difference in the migration ability of both cell lines. However, miR-1270 overexpression notably inhibited the invasion capacity of both cell lines through a semi-permeable membrane (Figure 4B). These findings suggested that miR-1270 could attenuate the proliferation and invasion abilities of the osteoblast-like cell lines U2OS and SaOS-2, thus regulating their functions. Overall, the results above indicated that miR-1270 could regulate IRF8 expression, which could serve a crucial role in the function and development of osteoblasts and osteoclasts.

## 4. Discussion

Bioinformatics analysis, miR-1270 overexpression in circulating monocytes derived from women with OP, and literature screening revealed that IRF8 was the most promising target gene of miR-1270 [29]. Therefore, the current study aimed to uncover the potential mechanism of action underlying miR-1270 overexpression. In vitro experiments were carried out to evaluate the regulatory effect of miR-1270 on IRF8 expression in bone-derived cell lines. The results show that miR-1270 exhibited an inhibitory regulation of IRF8 expression through binding to its 3′-UTR. Furthermore, miR-1270 overexpression attenuated the proliferation and invasion of SaOS-2 and U2OS cells.

The human mature miR-1270 is encoded by the MIR1270 gene located at 19p12 (20,399,272–20,399,354 PB). It has been reported that this miRNA serves as a key player in several types of cancer, including osteosarcoma, brain cancer, and bladder cancer [12,31,32]. However, its role in regulating bone metabolism remains elusive.

IRF8, a member of the IRF family of transcription factors (IRF1–9), plays a critical role in bone metabolism and myeloid cell differentiation, immune responses, and gene transcription [33,34]. A previous study demonstrated that IRF8 was directly targeted by miR-1270 [27]. Another study revealed that IRF8 could suppress osteoclastogenesis by inhibiting the nuclear factor of activated T cells (NFATc1), an essential transcription factor during osteoclast differentiation [35].

The bone microenvironment is a complex system that involves the crosstalk between osteoclasts and osteoblasts [17,18,36]. It has been reported that molecular communication between these cell types occurs during bone remodeling [18,21,24,25,36]. Based on the above findings, the present study hypothesized that miRNAs generated by osteoclasts (e.g., miR-1270) can regulate the gene expression profile in nearby osteoblasts. A possible mechanism is that osteoclasts secrete microRNA-enriched exosomes, by which miR-1270 is transferred into osteoblasts to inhibit their function. In accordance with this proposed mechanism, the authors in [37] suggested that osteoclasts secrete miR-214-enriched exosomes and that miR-214 is transferred into osteoblasts to inhibit their bone-formation activity. Taken together, these findings suggest that the exosome-mediated transfer of microRNA plays a key role in the regulation of osteoblast activity.

On the other hand, emerging evidence has suggested that miR-1270 plays an essential role in the interferon pathway [35]. The current study results revealed that miR-1270 could bind and regulate IRF8 expression. In addition, miR-1270 overexpression could inhibit the proliferation and invasion of osteoblast-like cells. However, the decrease in the migration ability of osteoblast-like cells was not statistically significant, possibly due to the stage of cell differentiation [38,39]. 

Alterations in complex processes such as bone remodeling cannot result from changes in the expression of a single miRNA. Therefore, it is widely accepted that coordinating the expression of several molecules is necessary in order to verify their pathological effects on complex systems. In this regard, current studies mainly focus on the combined effects of miRNAs, proteins, and genetic alterations such as single-nucleotide polymorphisms on the development of OP. It is widely accepted that the crosstalk between osteoclasts and osteoblasts is a necessary mechanism for regulating the homeostasis of bone metabolism [11,40]. In turn, miRNAs play a crucial role in regulating the expression of genes associated with bone metabolism. Therefore, uncovering the effects of miRNAs on this complex cellular process is essential for a better understanding of OP, and for improving its diagnosis and treatment miR-1270 could act via harmonizing with previously identified miRNAs to modulate osteoblast differentiation and the development of OP. For this reason, exploring the function of different miRNAs on bone metabolism is of great importance.

## 5. Conclusions

The present study confirmed that miR-1270 was associated with bone metabolism. In addition, the results demonstrated that miR-1270 could modulate IRF8 expression and be involved in osteoblast differentiation, possibly due to a mechanism of exosome-mediated transfer of microRNA. In addition, miR-1270 overexpression attenuated osteoblast proliferation and invasion.

Although further studies are required to fully uncover the molecular mechanisms underlying the effects of miR-1270 and IRF8 on the pathogenesis of OP, the results of the present study suggest that both molecules could be used as potential biomarkers and therapeutic targets for OP. 

In the future, further studies are required to identify multiple novel genetic and regulatory factors involved in bone metabolism that could represent a fruitful approach to treating OP.

## Data Availability

The datasets used and analyzed during the current study are available from the corresponding author on reasonable request.
